# Supplementary material for: Carbon fractions in the world’s dead wood
Source: Nat Commun. 2021 Feb 9;12:889. doi: 10.1038/s41467-021-21149-9 (PMC7873216; doi:10.1038/s41467-021-21149-9)
Supplement: Supplementary file 1 — Supplementary Information. [file 41467_2021_21149_MOESM1_ESM.pdf]

Supplementary Information for:

Carbon fractions in the world's dead wood

Authors and Affiliations:

Adam R. Martin[1,*], Grant M. Domke[2], Mahendra Doraisami[1], Sean C. Thomas[3]

[1] Department of Physical and Environmental Sciences, University of Toronto Scarborough, Canada.

[2] USDA Forest Service, Northern Research Station, St. Paul, Minnesota, USA

[3] Institute of Forestry and Conservation, Daniels Faculty of Architecture, Landscape, and Design, University of Toronto, Toronto, Ontario, Canada

Corresponding author contact information:

Email: adam.martin@utoronto.ca, Phone: 001-647-607-7058

Running header: Carbon concentration in dead wood

Keywords: Carbon accounting, coarse woody material, forest, greenhouse gas inventory, tree, wood carbon, wood trait, wood chemistry.

Supplementary Table 1. Analysis of variance (ANOVA) evaluating dead wood carbon fractions (CF) as a function of primary factors and all two-way interactions. Sample size for ANOVA model is n=973, and significant factors are highlighted in bold (where $p \leq 0.05$).

Factor	D.f.	Sum Sq.	Mean Sq.	*F* value	*p*
**Biome**	**3**	**1967**	**655.6**	**114.8**	**<0.001**
**Division**	**1**	**1120**	**1119.7**	**196.0**	**<0.001**
**Tissue type**	**4**	**636**	**158.9**	**27.8**	**<0.001**
Position	1	9	9.2	1.6	0.205
**DC**	**4**	**202**	**50.6**	**8.9**	**<0.001**
**Biome * division**	**2**	**180**	**89.9**	**15.7**	**<0.001**
**Biome * tissue**	**5**	**150**	**30.1**	**5.3**	**<0.001**
Biome * position	3	16	5.3	0.9	0.423
**Biome * DC**	**12**	**674**	**56.2**	**9.8**	**<0.001**
**Division*** tissue	3	19	6.4	1.1	0.343
**Division*** position	1	0	0.1	0.02	0.885
**Division*** DC	4	13	3.2	0.6	0.691
**Tissue * position**	**2**	**62**	**30.9**	**5.4**	**0.005**
Tissue * DC	13	40	3.1	0.5	0.899
Position * DC	4	17	4.4	0.8	0.55
Residuals	910	5199	5.7		

Supplementary Table 2. Variance partitioning of dead wood carbon fractions (CF) in trees across six nested factors. Variance components are based on the method developed by Messier et al. (2010), with lower and upper 95% confidence limits generated through bootstrapping. Results are presented for two different nested structures.

Dataset	Factor	Variance explained	Lower C.I.	Upper C.I.
Full dataset with decay class nested within position.	Biome	0.23	0.161	0.303
	Division	0.076	0.033	0.141
	Tissue	0.189	0.078	0.279
	Position	0.002	0.0	0.047
	Decay class	0.088	0.054	0.223
	Unexplained	0.414	0.285	0.458
Full dataset with position nested within decay class.	Biome	0.228	0.155	0.302
	Division	0.070	0.03	0.131
	Tissue	0.181	0.071	0.273
	Decay class	0.083	0.0	0.225
	Position	0.022	0.01	0.183
	Unexplained	0.417	0.28	0.471

Supplementary Table 3. Analysis of variance for five separate mixed-effects models, predicting dead wood carbon fractions (CFs) as a function of five different factors. The models presented here form the basis of the least squares mean CF values presented in Table 1 and Figure 2 of the main text.

**Model**	**Sum Sq.**	**Mean Sq.**	**Num. D.F.**	**Den. D.F.**	***F***	**Model *p***
Biome	542.34	180.78	3	928.1	28.314	<0.001
Division	583.54	583.54	1	937.29	101.38	<0.001
Tissue	301.63	75.407	4	932.01	13.029	<0.001
Position	22.1	22.1	1	924.59	3.9779	0.046
Decay class	180.79	45.197	4	953.25	7.2932	<0.001

Supplementary Table 4. Species-specific sample sizes for dead wood CF observations across decay classes (DCs). Highlight in bold are species with dead wood CF data for at least four DCs, which were then included in subsequent analyses on dead wood CF changes through decomposition (see Figure 3 in main text, and Supplementary Table 5).

	Sample Sizes					
**Species**	**DC 1**	**DC 2**	**DC 3**	**DC 4**	**DC 5**	**Total**
Abies alba	**1**	**1**	**1**	**1**	**1**	**5**
Abies amabilis	2	2	0	0	0	4
Abies balsamea	**3**	**2**	**3**	**1**	**3**	**12**
Abies concolor	**6**	**4**	**4**	**4**	**4**	**22**
Abies grandis	2	2	2	0	0	6
Abies lasiocarpa	**6**	**2**	**3**	**1**	**1**	**13**
Abies magnifica	**5**	**5**	**2**	**4**	**2**	**18**
Abies procera	0	2	2	2	0	6
Acacia auriculaeformis	1	0	0	0	0	1
Acacia polyphylla	0	0	1	0	0	1
Acer rubrum	2	2	1	0	0	5
Albizia polycephala	1	0	0	0	0	1
Allophylus edulis	1	0	0	0	0	1
Alnus glutinosa	**1**	**1**	**1**	**1**	**1**	**5**
Alnus incana	**1**	**1**	**1**	**1**	**1**	**5**
Alnus rubra	**4**	**4**	**2**	**2**	**1**	**13**
Altingia gracilipes	**1**	**1**	**1**	**1**	**1**	**5**
Anadenanthera peregrina var falcata	0	0	1	0	0	1
Aphanamixis polystachya	1	0	0	0	0	1
Aspidosperma polyneuron	0	0	1	0	0	1
Betula papyrifera	**1**	**2**	**2**	**1**	**2**	**8**
Betula pendata	**4**	**4**	**4**	**4**	**2**	**18**
Betula pendula	**4**	**4**	**4**	**4**	**4**	**20**
Betula pubescens	**1**	**1**	**1**	**1**	**1**	**5**
Brosimum alicastrum	0	1	1	1	0	3
Bursera simaruba	**2**	**2**	**2**	**2**	**0**	**8**
Calocedrus decurrens	**2**	**4**	**4**	**3**	**2**	**15**
Carallia brachiate	1	0	0	0	0	1

Carya alba	2	2	2	0	0	6
***Carya* sp.**	**0**	**2**	**1**	**1**	**1**	**5**
Castanopsis carlesii	**1**	**1**	**1**	**1**	**1**	**5**
Castanopsis chinensis	1	2	1	0	0	4
Castanopsis faberi	**0**	**1**	**1**	**1**	**1**	**4**
Cecropia obtusifolia	**2**	**1**	**1**	**1**	**0**	**5**
Centrolobium tomentosum	0	0	1	0	0	1
Cestrum sp.	1	0	0	0	0	1
Chamaecyparis japonica	**1**	**1**	**1**	**1**	**0**	**4**
Chamaecyparis obtusa	**1**	**1**	**1**	**1**	**0**	**4**
Cinnamomum chekiangense	**1**	**1**	**1**	**1**	**1**	**5**
Copaifera langsdorffii	0	0	1	0	0	1
Croton floribundus	**0**	**1**	**1**	**1**	**1**	**4**
Cryptocarya concinna	1	2	1	0	0	4
Cupania vernalis	1	0	0	0	0	1
Dalbergia glomerata	2	0	2	0	0	4
Dendropanax arboreus	0	1	1	0	0	2
Dialium guineense	0	0	1	0	0	1
Esenbeckia leiocarpa	0	0	1	0	0	1
Fagus crenata	0	41	0	0	0	41
Fagus grandifolia	2	2	1	0	0	5
Fagus sylvatica	**3**	**3**	**3**	**3**	**1**	**13**
Ficus yoponensis	2	0	2	0	0	4
Heliocarpus appendiculatus	**2**	**1**	**1**	**1**	**0**	**5**
Inga striata	0	0	1	0	0	1
Larix sibirica	**4**	**4**	**4**	**2**	**2**	**16**
Liquidambar styraciflua	2	2	2	0	0	6
Liriodendron tulipifera	**4**	**4**	**2**	**1**	**0**	**11**
Luehea divaricata	1	0	0	0	0	1
Machaerium brasiliense	0	0	1	0	0	1
Magnolia odora	**1**	**1**	**1**	**1**	**1**	**5**
Manilkara zapota	2	0	2	0	0	4
Matayba elaeagnoides	1	0	0	0	0	1
Melicoccus oliviformis	0	1	1	0	0	2
Myrcianthes fragrans	0	1	1	0	0	2
Nectandra ambigens	**2**	**1**	**1**	**1**	**0**	**5**

Nectandra globosa	0	1	0	1	0	2
Nectandra lanceolata	1	0	0	0	0	1
Nectandra megapotamica	1	0	0	0	0	1
Nothofagus antarctica	0	4	0	0	0	4
Nothofagus betuloides	0	4	0	0	0	4
Nothofagus pumilio	0	4	0	0	0	4
***Ocotea* sp.**	**0**	**1**	**1**	**1**	**1**	**4**
Orthion oblanceolatum	0	0	1	0	0	1
Parapiptadenia rigida	1	0	0	0	0	1
Picea abies	**21**	**17**	**15**	**15**	**10**	**78**
Picea engelmannii	2	0	1	1	0	4
Picea lutzii	**4**	**4**	**4**	**2**	**2**	**16**
Picea sitchensis	**2**	**2**	**2**	**2**	**2**	**10**
***Picea* sp.**	**0**	**1**	**1**	**1**	**1**	**4**
Pinus armandi	**1**	**1**	**1**	**1**	**1**	**5**
Pinus banksiana	**5**	**6**	**6**	**3**	**2**	**22**
Pinus contorta	**6**	**4**	**5**	**1**	**1**	**17**
Pinus echinata	**2**	**4**	**2**	**1**	**0**	**9**
Pinus jeffreyi	**2**	**2**	**1**	**1**	**0**	**6**
Pinus lambertiana	**4**	**4**	**4**	**4**	**2**	**18**
Pinus monticola	2	2	2	0	0	6
Pinus ponderosa	**4**	**4**	**4**	**4**	**4**	**20**
Pinus radiata	6	6	3	0	0	15
Pinus resinosa	**4**	**1**	**5**	**2**	**4**	**16**
Pinus siberica	**4**	**4**	**2**	**2**	**2**	**14**
Pinus sylvestris	**10**	**10**	**7**	**7**	**4**	**38**
Pinus taeda	**4**	**4**	**3**	**2**	**1**	**14**
Piptadenia gonoacantha	0	0	1	1	0	2
Piptocarpha sellowii	0	0	1	0	0	1
Populus tremula	**1**	**1**	**1**	**1**	**1**	**5**
Populus tremula x Populus tremuloides	1	0	0	0	0	1
Populus tremuloides	**5**	**6**	**6**	**3**	**2**	**22**
Prunus brasiliensis	1	0	0	0	0	1
Pseudolmedia glabrata	**2**	**2**	**2**	**2**	**2**	**10**
Pseudotsuga menziesii	**9**	**6**	**9**	**8**	**8**	**40**
Qualea sp.	0	0	1	0	0	1

Quercus crispula	0	38	0	0	0	38
Quercus alba	**2**	**2**	**2**	**1**	**0**	**7**
Quercus aliena var acuteserrata	**1**	**1**	**1**	**1**	**1**	**5**
Quercus rubra	**2**	**2**	**2**	**1**	**0**	**7**
Quercus species	0	0	1	1	0	2
Quercus velutina	**2**	**1**	**6**	**4**	**5**	**18**
***Sabal* sp.**	**2**	**2**	**2**	**2**	**0**	**8**
Savia dictyocarpa	0	0	1	1	0	2
Schefflera heptaphylla	1	0	0	0	0	1
Schima superba	1	2	1	0	0	4
Sciadopitys verticillata	1	0	0	0	0	1
Sideroxylon portoricense subsp minutiflorum	0	0	1	0	0	1
Simarouba amara	2	2	0	1	0	5
Swartzia cubensis	2	2	2	0	0	6
Swietenia macrophylla	0	2	2	0	0	4
Tapirira guianensis	0	1	1	1	0	3
Thuja plicata	**4**	**4**	**4**	**3**	**3**	**18**
Tsuga heterophylla	**8**	**8**	**8**	**8**	**2**	**34**
Vitex megapotamica	1	0	0	0	0	1
Vochysia tucanorum	0	0	1	0	0	1
Zanthoxylum rhoifolium	1	0	0	0	0	1

Supplementary Table 5. Model parameters describing species-specific changes in dead wood CFs as a function of decay class (DC). Models are presented graphically in Figure 3. Only species with wood CF from at least four decay classes are included in the analyses presented here (highlighted in Supplementary Table 4).

Species characteristics			Intercept			Slope		
**Species**	**Division**	**Biome**	**Estimate**	**S.E.**	***p*-value**	**Estimate**	**S.E.**	***p*-value**
Alnus glutinosa	Ang.	Boreal	48.76	2.60	<0.001	0.26	0.66	0.68
Alnus incana	Ang.	Boreal	46.97	2.60	<0.001	0.72	0.66	0.27
Betula papyrifera	Ang.	Boreal	49.45	2.30	<0.001	0.16	0.55	0.76
Betula pendata	Ang.	Boreal	44.46	1.78	<0.001	1.16	0.38	<0.001
Betula pendula	Ang.	Boreal	48.53	1.74	<0.001	0.33	0.33	0.31
Betula pubescens	Ang.	Boreal	46.17	2.60	<0.001	0.70	0.66	0.29
Populus tremula	Ang.	Boreal	48.08	2.60	<0.001	0.03	0.66	0.95
Populus tremuloides	Ang.	Boreal	49.20	1.63	<0.001	-0.03	0.38	0.92
Abies balsamea	Con.	Boreal	50.12	1.88	<0.001	0.13	0.40	0.75
Larix sibirica	Con.	Boreal	51.78	1.78	<0.001	-0.90	0.40	0.02
Picea abies	Con.	Boreal	48.87	1.42	<0.001	0.07	0.17	0.65
Picea sp.	Con.	Boreal	48.41	3.73	<0.001	0.74	0.94	0.43
Pinus banksiana	Con.	Boreal	50.25	1.70	<0.001	0.41	0.37	0.26
Pinus siberica	Con.	Boreal	50.97	1.79	<0.001	-0.08	0.40	0.84
Pinus sylvestris	Con.	Boreal	49.41	1.52	<0.001	0.22	0.25	0.37
Alnus rubra	Ang.	Temperate	50.03	1.78	<0.001	-0.38	0.46	0.40
Carya sp.	Ang.	Temperate	47.45	3.04	<0.001	0.11	0.81	0.88
Fagus sylvatica	Ang.	Temperate	48.12	1.85	<0.001	0.33	0.47	0.48
Liriodendron tulipifera	Ang.	Temperate	48.25	1.95	<0.001	0.03	0.67	0.96
Quercus alba	Ang.	Temperate	47.73	2.32	<0.001	-0.26	0.77	0.73
Quercus aliena var. acuteserrata	Ang.	Temperate	49.70	2.55	<0.001	1.11	0.66	0.09
Quercus rubra	Ang.	Temperate	45.92	2.32	<0.001	0.82	0.77	0.29
Quercus velutina	Ang.	Temperate	48.47	1.99	<0.001	0.73	0.40	0.07
Abies concolor	Con.	Temperate	48.79	1.66	<0.001	0.97	0.30	0.00
Abies lasiocarpa	Con.	Temperate	50.93	1.74	<0.001	-0.07	0.45	0.86
Abies magnifica	Con.	Temperate	49.59	1.71	<0.001	0.67	0.36	0.06
Calocedrus decurrens	Con.	Temperate	50.86	1.94	<0.001	0.27	0.44	0.53

Chamaecyparis japonica	Con.	Temperate	50.45	2.91	<0.001	0.46	0.94	0.62
Chamaecyparis obtusa	Con.	Temperate	51.95	2.91	<0.001	0.35	0.94	0.71
Picea lutzii	Con.	Temperate	50.66	1.78	<0.001	0.03	0.40	0.93
Picea sitchensis	Con.	Temperate	50.84	2.06	<0.001	-0.13	0.47	0.78
Pinus armandi	Con.	Temperate	53.98	2.59	<0.001	0.74	0.66	0.26
Pinus contorta	Con.	Temperate	49.39	1.73	<0.001	0.82	0.44	0.06
Pinus echinata	Con.	Temperate	49.94	2.28	<0.001	0.23	0.77	0.75
Pinus jeffreyi	Con.	Temperate	48.07	2.37	<0.001	1.29	0.81	0.11
Pinus lambertiana	Con.	Temperate	51.13	1.77	<0.001	0.23	0.38	0.53
Pinus ponderosa	Con.	Temperate	51.40	1.74	<0.001	0.16	0.33	0.63
Pinus resinosa	Con.	Temperate	49.48	1.87	<0.001	0.48	0.36	0.17
Pinus taeda	Con.	Temperate	49.95	1.83	<0.001	0.74	0.45	<0.001
Pseudotsuga menziesii	Con.	Temperate	49.61	1.54	<0.001	0.81	0.23	<0.001
Thuja plicata	Con.	Temperate	46.14	1.75	<0.001	1.63	0.36	<0.001
Tsuga heterophylla	Con.	Temperate	48.70	1.58	<0.001	0.89	0.29	<0.001
Altingia gracilipes	Ang.	Sub./Med.	51.74	2.67	<0.001	-2.12	0.66	<0.001
Castanopsis carlesii	Ang.	Sub./Med.	53.94	2.67	<0.001	-3.30	0.66	<0.001
Castanopsis faberi	Ang.	Sub./Med.	55.26	3.78	<0.001	-2.75	0.94	<0.001
Cinnamomum chekiangense	Ang.	Sub./Med.	56.20	2.67	<0.001	-4.14	0.66	<0.001
Croton floribundus	Ang.	Sub./Med.	54.94	3.78	<0.001	-0.58	0.94	0.54
Magnolia odora	Ang.	Sub./Med.	56.92	2.67	<0.001	-4.02	0.66	<0.001
Ocotea sp.	Ang.	Sub./Med.	53.82	3.78	<0.001	-0.21	0.94	0.82
Abies alba	Con.	Sub./Med.	44.85	3.14	<0.001	0.31	0.66	0.64
Bursera simaruba	Ang.	Tropical	43.90	2.68	<0.001	0.55	0.66	0.40
Cecropia obtusifolia	Ang.	Tropical	45.91	2.81	<0.001	0.26	0.82	0.74
Heliocarpus appendiculatus	Ang.	Tropical	45.02	2.81	<0.001	0.89	0.82	0.27
Nectandra ambigens	Ang.	Tropical	48.42	2.81	<0.001	0.30	0.82	0.70
Pseudolmedia glabrata	Ang.	Tropical	44.73	2.51	<0.001	0.62	0.47	0.19
Sabal sp.	Ang.	Tropical	48.35	2.68	<0.001	-0.48	0.66	0.46

Supplementary Table 6. Analysis of covariance model predicting dead wood carbon fractions (CFs) within a species, as a function of live wood CF, division (i.e., gymnosperm vs. angiosperm), as well as a live wood CF-by-division interaction term. Significant terms are highlighted in bold, and sample size was *n*=63 species (*n*=42 and *n*=21 angiosperms and gymnosperms, respectively). Model parameters are presented in Supplementary Table 7 and graphically in Supplementary Figure 1.

Variable	D.F.	Sum Sq.	Mean Sq.	*F*	*p*
**Live wood CF**	**1**	**299.6**	**299.6**	**72.3**	**<0.001**
**Division**	**1**	**91.2**	**91.2**	**22.0**	**<0.001**
Live CF*division	1	0.8	0.8	0.2	0.669
Residuals	59	244.6	4.2		

Supplementary Table 7. Parameters of a linear model predicting dead wood carbon fractions (CFs) within a species, as a function of live wood CF and division (i.e., gymnosperm vs. angiosperm). In this model, a live wood CF-by-division interaction term was not included (based on results presented in Supplementary Table 6), so only the intercept differs across species types. Significant terms are highlighted in bold, and sample size was *n*=63 species (*n*=42 and *n*=21 angiosperms and gymnosperms, respectively). Type-specific models are presented in Supplementary Figure 1.

Parameter	Estimate	S.E.	*t*	*p*
**Intercept**	**27.6**	**4.9**	**5.7**	**<0.001**
**Live wood CF**	**0.4**	**0.1**	**3.8**	**<0.001**
**Gymnosperm intercept term**	**3.3**	**0.7**	**4.7**	**<0.001**

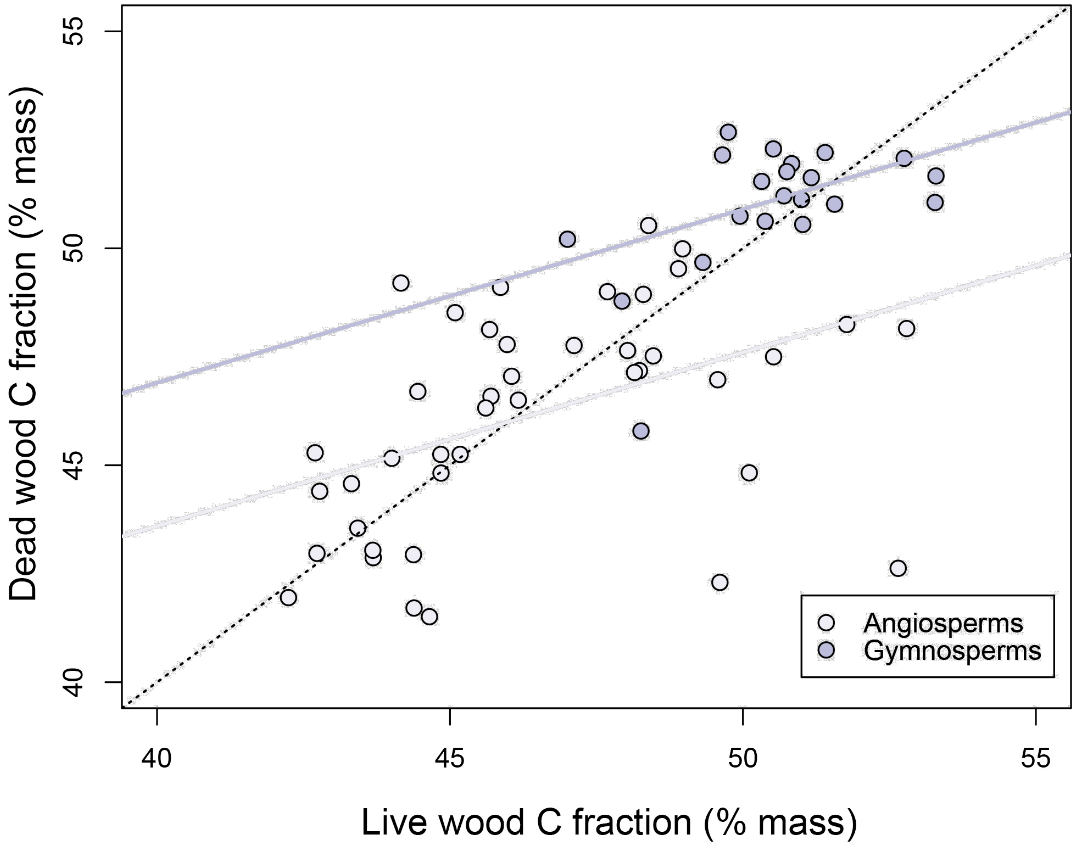

Supplementary Figure 1. Dead wood C fractions as a function of live wood C fractions across 42 angiosperm and 21 gymnosperm tree species. Solid lines corresponds to a linear model fit based on analysis of covariance (Supplementary Table 7) for angiosperms and gymnosperms, and the black dotted line represents a 1:1 relationship.
